# The national inventory of core capabilities for pandemic influenza preparedness and response: an instrument for planning and evaluation

**DOI:** 10.1111/irv.12218

**Published:** 2013-12-23

**Authors:** Goldie MacDonald, Ann C Moen, Michael E St Louis

**Affiliations:** a Center for Global Health, Centers for Disease Control and PreventionAtlanta, GA, USA; b National Center for Immunization and Respiratory Diseases, Centers for Disease Control and PreventionAtlanta, GA, USA

**Keywords:** Evaluation, pandemic influenza, planning, preparedness

## Abstract

**Background:**

Reviews of the global response to the 2009 pandemic of influenza A/H1N1 affirmed the importance of assessment of preparedness and response capabilities.

**Design:**

The U. S. Centers for Disease Control and Prevention (CDC) and partners developed the National Inventory of Core Capabilities for Pandemic Influenza Preparedness and Response (http://www.cdc.gov/flu/international/tools.htm) to collect data on coverage, quality, and timeliness in 12 domains: country planning, research and use of findings, communications, epidemiologic capability, laboratory capability, routine influenza surveillance, national respiratory disease surveillance and reporting, outbreak response, resources for containment, community-based interventions to prevent the spread of influenza, infection control, and health sector pandemic response. For each of the capabilities, we selected four indicators. Each indicator includes four levels of performance (0–3), ranging from no or limited capability to fully capable.

**Results:**

In 2008, 40 countries in 6 regions of the World Health Organization (WHO) collected data using the instrument. In 2010 and 2012, 36 and 39 countries did so, respectively. Data collection at regular intervals allows changes in preparedness and response capabilities to be documented. In most countries, participants used the instrument and data collected to inform discussion and planning toward improving the country's level of preparedness for pandemic influenza.

**Conclusions:**

The National Inventory provides countries with a systematic method to document the status of their capabilities with regard to pandemic influenza and to assess progress over time. The National Inventory produces data and findings that serve a wide range of users and uses.

## Introduction

In the context of pandemic influenza, preparedness includes… ‘the capability of the public health and health care systems, communities, and individuals, to prevent, protect against, quickly respond to, and recover from health emergencies, particularly those whose scale, timing, or unpredictability threatens to overwhelm routine capabilities’.^1^ As such, many countries developed and continue to implement national influenza pandemic preparedness plans.^2^ The content and use of these plans has been reviewed in a number of studies.^3^–^5^ Yet, reviews of the functioning of the International Health Regulations in relation to the 2009 pandemic of influenza A/H1N1 concluded that the world is not prepared for a severe influenza pandemic.^6^ The World Health Organization (WHO) recommends that each country assess its preparedness status and identify immediate steps needed to fill gaps.^7^ The assessment of capabilities relevant to preparedness for pandemic influenza remains a priority. The U. S. Centers for Disease Control and Prevention (CDC) and partners developed the National Inventory of Core Capabilities for Pandemic Influenza Preparedness and Response (http://www.cdc.gov/flu/international/tools.htm) to address this need.

## Discussion

### Overview of the instrument

The National Inventory of Core Capabilities for Pandemic Influenza Preparedness and Response (National Inventory) includes the assessment of indicators or measures of coverage, quality, and timeliness in 12 domains of pandemic influenza preparedness and response: country planning, research and use of findings, communications, epidemiologic capability, laboratory capability, routine influenza surveillance, national respiratory disease surveillance and reporting, outbreak response, resources for containment, community-based interventions to prevent the spread of influenza, infection control, and health sector pandemic response. For each of the twelve capabilities, we selected four indicators. Each indicator is divided into four levels of performance (0–3), ranging from no or limited capability to fully capable. These capabilities and indicators are limited to those related to human health.

As individual countries and international organizations work to strengthen preparedness and response capabilities, the National Inventory can be used to document a country's capabilities at a point in time, determine its progress toward improved preparedness, demonstrate accountability for use of resources to donors and stakeholders, guide ongoing investment in preparedness and response, inform planning across pandemic phases, show logistic and operational needs and consider how to address deficiencies according to available resources, promote exchange of information within a country, and support harmonization of national and international preparedness and response activities.

### Development of the national inventory

The National Inventory is based on a set of guiding principles – assumptions or decisions made in conjunction with stakeholders that inform the instrument's design and implementation (Table 1). Each principle established plain-language parameters for the activity. CDC and its partners created an instrument that would provide accurate, comparable information on levels of preparedness and response across countries and over time. To do so, we paid attention to both the instrument's architecture and its content. The National Inventory assesses 12 capabilities comprising 4 indicators for each capability (Table 2). For each capability, we created a matrix with a description of each of its indicators and a description of each indicator's four levels of performance (0 – 3), which range from none or limited to fully capable. See Table 3 for an example of the matrix for one capability. The National Inventory allows users to see the indicators as progressive and relational (i.e., users can see the level of capability as a product of the indicators collectively or in relation to each other). The score for each capability is the average of the scores for the four indicators; to determine the level of preparedness for each capability, we do not rely on a single indicator to define or represent the capability. Although many countries continue to work toward improving their level of preparedness, the aims of each country are not identical. Given the ongoing, widespread discussion about the evidence base for the best way to prepare for, and respond to an influenza pandemic, questions remain as to the most informative indicators of a country's or region's capabilities.

**Table 1:** Guiding principles for development and implementation of the national inventory of core capabilities for pandemic influenza preparedness and response

• The National Inventory is based on the best available evidence and practice-wisdom relevant to preparedness for, and response to, pandemic influenza.
• Data collected indicate composite progress in specific – not all – capabilities of preparedness and response relevant to pandemic influenza.
• Data collection should not impose unnecessary burden on participating countries or partner organizations.
• Progress from one level to the next demonstrates a meaningful improvement in public health function.
• Although all countries work to improve their level of preparedness, the objectives of all countries need not be identical.
• Full achievement of these capabilities does not guarantee that an emerging pandemic will be interrupted.

**Table 2:** The national inventory of core capabilities for pandemic influenza preparedness and response – 12 areas of capability to be evaluated in each country

Capability	Indicators
Capability 1: Country planning	Status of plan Dissemination Exercises Coordination and resources for the implementation of country plan
Capability 2: Research and use of findings	Collaboration between human and animal health Research priorities Environment of support for research and use of findings Use of data to inform decisions for pandemic influenza preparedness
Capability 3: Communications	Status of communications plan Messaging Dissemination Staffing
Capability 4: Epidemiologic capability	Operational status Epidemiologists and field epidemiologists Quality of public health epidemiologists Training
Capability 5: Laboratory capability	National influenza laboratory network Biosafety level (BSL) and routine testing of specimens Methods Participation in the World Health Organization (WHO) system
Capability 6: Routine influenza surveillance	Integration of virologic and epidemiologic surveillance Data publication Timeliness Case definitions
Capability 7: National respiratory disease surveillance and reporting	Awareness of need to report Rumor reporting and media scanning Cross-notification Timeliness
Capability 8: Outbreak response	Human resources for outbreak response Logistical resources for outbreak response Exercises or response Activation of team
Capability 9: Resources for containment	Availability of antivirals Storage facilities Exercises and practice Distribution of materials
Capability 10: Community-based interventions to prevent the spread of influenza	Social distancing Critical infrastructure Voluntary isolation and quarantine Percent of districts with plan
Capability 11: Infection control	Standards of infection control by level of the healthcare system Human resources Logistical resources Institutionalization of infection control efforts
Capability 12: Health sector pandemic response	Surge capacity – human resources Surge capacity – physical facilities and equipment Clinical management guidelines Surge capacity – care of deceased

**Table 3:** The national inventory of core capabilities for pandemic influenza preparedness and response – capability 5: laboratory capability

Capability 5: Laboratory capability	Level of capability			
				Advanced
	0	1	2	3
Indicators				
(A) National Influenza Laboratory Network	No or limited planning for laboratory for testing of influenza	National laboratory for testing of influenza	National laboratory with one or more subnational laboratories sending specimens for testing or confirmation	National laboratory that routinely returns results of testing to referring laboratories
(B) Biosafety level (BSL) and routine testing of specimens	No or limited planning for laboratory for testing of influenza	National laboratory with biosafety level 2; does not routinely test influenza specimens	National laboratory with biosafety level 2; routinely tests influenza specimens; participates in WHO External Quality Assurance Project (EQAP)	National laboratory with biosafety of at least level 3; able to isolate avian influenza in humans
(C) Methods	No testing or identify influenza virus using rapid tests	Identify seasonal influenza virus, type and subtype; identify novel influenza viruses using molecular techniques	Isolate seasonal influenza virus, type and subtype using hemagglutination inhibition test	Full antigenic and genetic characterization of influenza viruses; isolate novel influenza viruses under biosafety level 3+
(D) Participation in who system	No or limited reporting to WHO; planning or preparation to comply with International Health Regulations (IHR)	Working toward fulfilling terms of reference for a National Influenza Center; regularly reports to WHO; shares specimens and/or isolates with WHO	Established National Influenza Center; actively reports through FluNet; routinely shares specimens and/or isolates for seasonal and avian influenza	Actively reports and shares results with WHO within 48 hours of laboratory confirmation of a potential Public Health Emergency of International Concern (PHEIC)

### Selecting core capabilities and indicators

A country's level of preparedness for pandemic influenza depends on its capability to engage in a range of activities during each of the pandemic phases. We selected indicators that would provide a good estimate of a country's level of preparedness, but these indicators do not represent the full scope of activities involved in preparing for, or responding to, a pandemic. Instead, each indicator provides a reasonable proxy or measure of preparedness or response capability. Each indicator speaks to some dimension of coverage, quality, or timeliness in preparedness or response. To select the final set of capabilities and the indicators we would use as proxies for those capabilities, a small group of CDC staff and partners first produced a preliminary list of relevant capabilities and levels of performance at a meeting in South-East Asia in 2006. We next reviewed the published literature and guidance documents related to pandemic influenza and extracted information on the key elements discussed in each document. We revised our list of capabilities and began to select indicators for each capability. The capabilities and indicators included, but were not limited to, U.S. Department of Health and Human Services (HHS) and CDC areas of investment and programmatic priorities. We identified 10 criteria by which we would judge each indicator: strength of evidence to support use of the indicator, utility of the information to the country, face validity, accepted practice or use of the indicator in other assessments, availability of data, data quality, investment of resources, burden of data collection on participants, and applicability in different countries or regions of the world.

We revised the capabilities and indicators on the basis of feedback from subject matter experts, national and international partners, and other stakeholders. All stakeholders agreed that the levels of performance selected for each indicator must show a meaningful improvement in a country's preparedness or response functions. We cross-referenced the selected levels of performance with the World Health Organization (WHO) Checklist for influenza pandemic preparedness planning^8^ and other relevant documents. While the National Inventory addresses many of the same topics presented in these resources (e.g., communication processes and protocols, surveillance activities), the content is further operationalized for measurement in the form of indicators and levels of performance. We pilot-tested the capabilities and indicators in three countries, each in a different region of WHO. On the basis of pilot test results, we made several changes: We added a capability and four indicators in order to more accurately document the work of countries in preparedness and response within the health sector (e.g., hospitals); we prepared detailed notes for each indicator to clarify and further operationalize the content; and we decided that data should be collected on paper rather than electronically to allow facilitators and participants to focus on discussion of the capabilities and indicators (as opposed to the technology used to collect data). The resulting instrument (National Inventory) includes 12 capabilities with 48 indicators and 4 levels of performance for each indicator.

### Implementing the national inventory

The implementation protocol requires that data collectors perform certain tasks before, during, and following the assessment. The protocol also recommends the type of participants needed for data collection and provides instructions for facilitators. In addition, the instrument has a page of notes for each indicator to inform the discussion with participants. These notes include the primary question to be asked for each capability, definitions of terms relevant to the capability or indicators, examples of sources of information or documentation relevant to the indicators, and references to support the indicators as important components of preparedness and response. In many cases, stakeholders consider that the process of determining the level of preparedness is as important as the results (i.e., level or score). Although all participants in data collection in each country work on some aspect of preparedness or response, their discussions during data collection provide opportunities for parties who do not always work together to collaborate on the evaluation. Collecting data and preparing a report on findings takes 1–2 days. When the report is complete, the facilitator validates the collected data by providing participants the scores agreed on for each capability and indicator, as well as detailed comments or notes on achievements and activities in process toward achieving or maintaining a score. In addition, participants collaborate on determining which items need immediate action or follow-up. In the weeks after data collection, the country receives from the facilitator a report that includes scores for each capability and indicator, documentation or evidence for each score, and notes that provide context for the scores.

## Results

In 2008, 40 countries in 6 WHO regions collected data using the National Inventory. In 2010 and 2012, 36 and 39 countries did so, respectively. Because ease of use and data quality were high priorities for developers of the National Inventory, it was implemented successfully, and data used, in countries worldwide. Because preparedness is not a dichotomous state (i.e., prepared or not prepared), we need an estimate of a country's status that is as accurate as possible at a point in time. Using this set of indicators provides that estimate of preparedness in 12 domains. And continuing to collect data in subsequent years allows changes in preparedness and response capabilities to be documented. In addition, each indicator represents a pathway to improved preparedness and response capabilities that were not explicit in other documents. In most countries, participants used the instrument and data collected to consider which activities and investments would be best for improving the country's level of preparedness for pandemic influenza.

In each country, participants determined the score for each indicator through in-depth discussion guided by two trained facilitators. Using facilitators increased the quality of data collected because they established, for example, a consistent process across sites and standard definitions of indicators, which allow the data to be comparable across sites and over time. The facilitators were also a resource for participants in each country: they could clarify text or explain how to implement the protocol. In addition, they checked the quality of data as it was being collected.

In-depth discussion among participants about each indicator yielded meaningful information beyond the numerical scores (e.g., items for action in the short term). As a result of the discussion, facilitators and participants saw a pathway to improved preparedness and response capabilities. Perhaps more importantly, using the National Inventory and discussing the country's findings helped to show officials how to move along that pathway informed by detailed information collected as a result of using the tool. Finally, the discussion brought together each country's key personnel who are involved in various aspects of pandemic influenza preparedness and response. During data collection, representatives from multiple government departments and agencies, as well as an array of external partners, shared achievements, described their current activities, and discussed challenges in their work to increase preparedness and response capabilities. The result was an increased awareness of the country's collective efforts to improve preparedness for pandemic influenza.

## Conclusions

The National Inventory provides countries with a practical, systematic method to document the status of their preparedness and response capabilities with regard to pandemic influenza and to assess progress over time. While the National Inventory can produce data and findings to serve a wide range of users and uses, the content is limited to the 12 areas of capability presented in Table 2. For example, the tool does not include capabilities or indicators relevant to production or use of vaccines to mitigate the impact of an influenza pandemic, or ethical and legal frameworks relevant to preparedness and response. However, the instrument is flexible and allows for the addition of new capabilities and indicators over time. Despite competing demands for resources during the most recent pandemic, many countries kept implementation of the National Inventory as a priority. As countries expand their preparedness and response capabilities, the National Inventory can serve as a resource-efficient instrument for planning and evaluation in diverse settings over time.
